# Risks associated with dispersive nocturnal flights of sylvatic Triatominae to artificial lights in a model house in the northeastern plains of Colombia

**DOI:** 10.1186/s13071-015-1209-3

**Published:** 2015-11-19

**Authors:** David Jácome-Pinilla, Eduwin Hincapie-Peñaloza, Mario I. Ortiz, Juan David Ramírez, Felipe Guhl, Jorge Molina

**Affiliations:** Centro de Investigaciones en Microbiología y Parasitología Tropical (CIMPAT), Departamento de Ciencias Biológicas, Universidad de los Andes, Bogotá, Colombia; Fundación Cataruben, Casanare-Bogotá, Colombia; Grupo de Investigaciones Microbiológicas Universidad del Rosario (GIMUR), Facultad de Ciencias Naturales y Matemáticas, Universidad del Rosario, Bogotá, Colombia; Facultad de Ciencias, Universidad de los Andes, A.A. 4976 Carrera 1a # 18A-10, Bogotá, Colombia

**Keywords:** *Rhodnius prolixus*, *Trypanosoma cruzi*, Chagas disease, Palm trees, Active dispersal, Light trap, Flight initiation

## Abstract

**Background:**

Control initiatives and continuous surveillance of vector-borne transmission have proved to be effective measures for diminishing the incidence of Chagas disease in endemic countries. However, the active dispersal of infected sylvatic adult triatomines by flight represents one of the main obstacles to eliminating domestic transmission.

**Methods:**

In order to determine the risk that active dispersal of sylvatic adult triatomines represents in Colombian northeastern plains, we quantified the distribution and abundance of triatomines in palm trees (primarily *Attalea butyracea*) using live bait traps. Directional light traps were used to estimate the frequency of sylvatic triatomine dispersal and their possible origin. Finally, the effect of environmental parameters and artificial light sources on the take-off of sylvatic *Rhodnius prolixus* was evaluated in field experiments.

**Results:**

*R. prolixus* was found in 90 % of the palm trees that densely aggregated toward the northern portion of the study area. *R. prolixus,* and three other sylvatic triatomine species were found to actively disperse and were attracted to the directional light traps (*Triatoma maculata*, *Panstrongylus geniculatus* and *Psammolestes arthuri*). Temperature, relative humidity, wind speed and night luminosity did not affect the active dispersal of the triatomines which is higher the first two hours after sunset. Artificial lights from houses at 60 and 110 m played a key role in the directionality of the *R. prolixus* take-offs. *Trypanosoma cruzi* was isolated from *R. prolixus, T. maculata* and *P. geniculatus* and was genotyped as *T. cruzi* I, III and IV.

**Conclusions:**

Our results highlight the potential risk in Colombian northeastern plains of actively dispersing sylvatic triatomines and their role in the domestic introduction of Discrete Typing Units of *T. cruzi* associated to sylvatic foci of Chagas disease transmission.

## Background

Chagas disease, caused by *Trypanosoma (Schyzotrypanum) cruzi* Chagas 1909*,* is a current public health problem in the Americas, with nearly 28 million people at risk of infection, 12,500 annual deaths and 41,200 new human cases each year due to vector transmission [1, 2]. This parasite displays remarkable genetic diversity as evidenced by at least six discrete typing units (DTUs). Intriguingly, the distribution of these DTUs is associated with domestic and sylvatic foci; TcI, TcII, TcV and TcVI are associated with the domestic cycle, and TcI, TcIII and TcIV are associated with sylvatic foci with sporadic cases in humans [3]. Triatomine bugs (Reduviidae: Triatominae), which are highly anthropophilic and are capable of establishing permanent colonies in human dwellings, are considered to be the vectors of *T. cruzi* [1, 4].

In Colombia, nearly a quarter of the population is at risk for *Trypanosoma cruzi* infection, and approximately 7 % are infected with the parasite [5]. Most of the vectorial transmission in Colombia is attributed to *Rhodnius prolixus* Stål 1859*,* because of its wide geographical distribution and its presence in domestic, peridomestic and sylvatic (associated with *Attalea* spp. and *Elaeis* spp. palm trees) habitats [5].

Strategies to control Chagas disease in Latin America have been based on the interruption of vector transmission by eliminating domestic insect populations with residual insecticides [6, 7]. However, several problems have emerged that have prevented a complete reduction in vectorial Chagas transmission, including the re-infestation of houses by sylvatic and peridomestic triatomines [8–11], colonization and re-infestation by species of triatomines that are considered to be secondary vectors [12–15] and the re-establishment of residual populations that were not entirely eliminated by chemical treatments [16].

Laboratory [17–19] and field studies [12, 13, 20–24] have proved the importance of sylvatic/peridomestic flying triatomine adults in the infestation and re-infestation of houses, except for a few cases reported with *Triatoma infestans* Klug 1834 [25, 26].

The role of artificial light sources in the attraction of actively dispersing triatomines has been tested [12, 13, 21, 27–33]. In addition to light, nutritional status, feeding rates during metamorphosis of the insects, environmental temperature and dimorphism of flight-related muscles have all been recognized for their roles in active dispersal [12, 17, 20, 21, 23, 27, 30, 34–40].

The relevance of active triatomine dispersal to Chagas disease transmission has led a committee of experts to recommend studies in Colombian and Venezuelan grassland plains in order to determine the epidemiological risk that sylvatic populations of *R. prolixus* represent in those areas [1]. In Colombia, the relevance of sylvatic triatomine species in Casanare and Arauca plains has recently been highlighted by the finding that *R. prolixus*, *Psammolestes arthuri* (Pinto, 1926), *Cavernicola pilosa* (Barber 1937), *Triatoma maculata* (Erichson, 1848) and *Panstrongylus geniculatus* (Latreille, 1811) are reaching human dwellings [41]. Due to this plethora of events, the aim of this study was to characterize the active flight dispersal of sylvatic triatomines in a typical house in Casanare grassland plains using two approaches: i) First, we quantified the presence and abundance of triatomines in palm trees around a house in the region. ii) Second, we quantified the number of triatomines that appeared to be attracted to light sources and to the house, and we attempted to correlate the flight dispersal with environmental parameters. Finally, we quantified the attractiveness of artificial light bulbs in take-off experiments with sylvatic *R. prolixus* that had previously been captured during active dispersal.

## Methods

### Study area and palm tree sampling

**Fig. 1:**
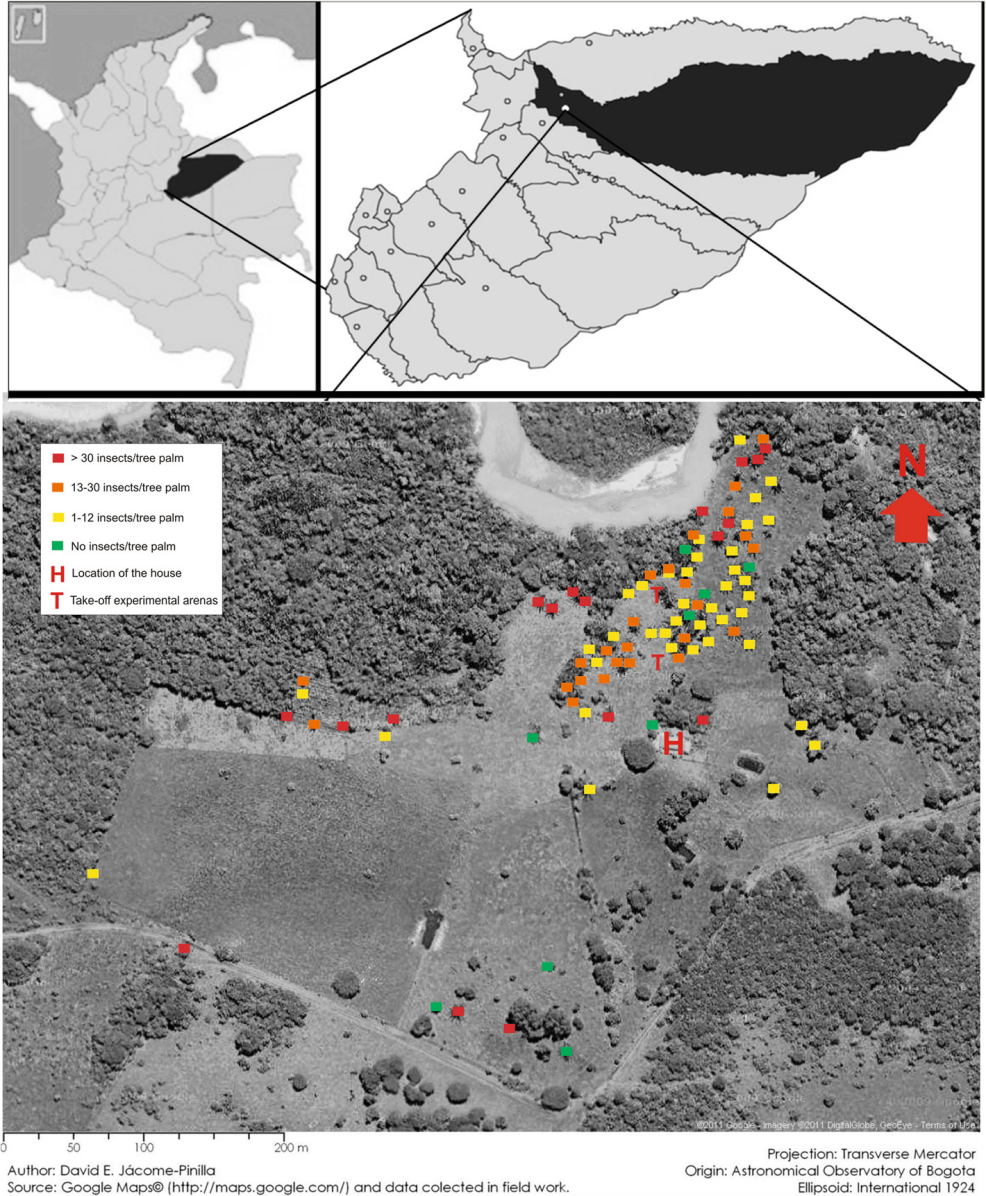
Study area and abundance of triatomines in palm trees. Map of the location showing with colors the distribution and abundance of sylvatic triatomines in palm trees at Miramar farm in Paz de Ariporo (Casanare-Colombia). Satellite image from Google Maps®

Fieldwork was carried out in Paz de Ariporo Municipality (Casanare) in northeast Colombia (5° 50’ 19”N, 71° 53’ 31”W and 360 m.a.s.l.). Prevalence of Chagas disease in Paz de Ariporo Municipality is 14.75 % considering both urban and rural areas, but in rural areas the prevalence is 22.22 % for females and 14.81 % for males [42]. The study area consisted of a house surrounded to the north by grassland plains with dispersed palm trees and gallery forests along the Muese river and to the south by grassland plains with few palm trees (Fig. 1). Ninety-four palm trees (eighty-eight *Attalea butyracea* (Mutis ex L.F.) Wess. Boer, five *Acrocomia aculeata* (Jacq.) Lood. ex Mart., and one *Cocos nucifera* Linnaeus) were sampled in the grassland plains around the house (Fig. 1). A modified live bait trap was used to capture triatomines in palm trees [43]. Each palm tree was sampled for one night with one live bait trap between 18:00 h and 6:00 h [44]. The geographical position of the palm tree and the number of insects, species, and nymphal stages captured were registered. All field work was carried out on private land with owners’ permission.

### Directional light trap experiments

**Fig. 2:**
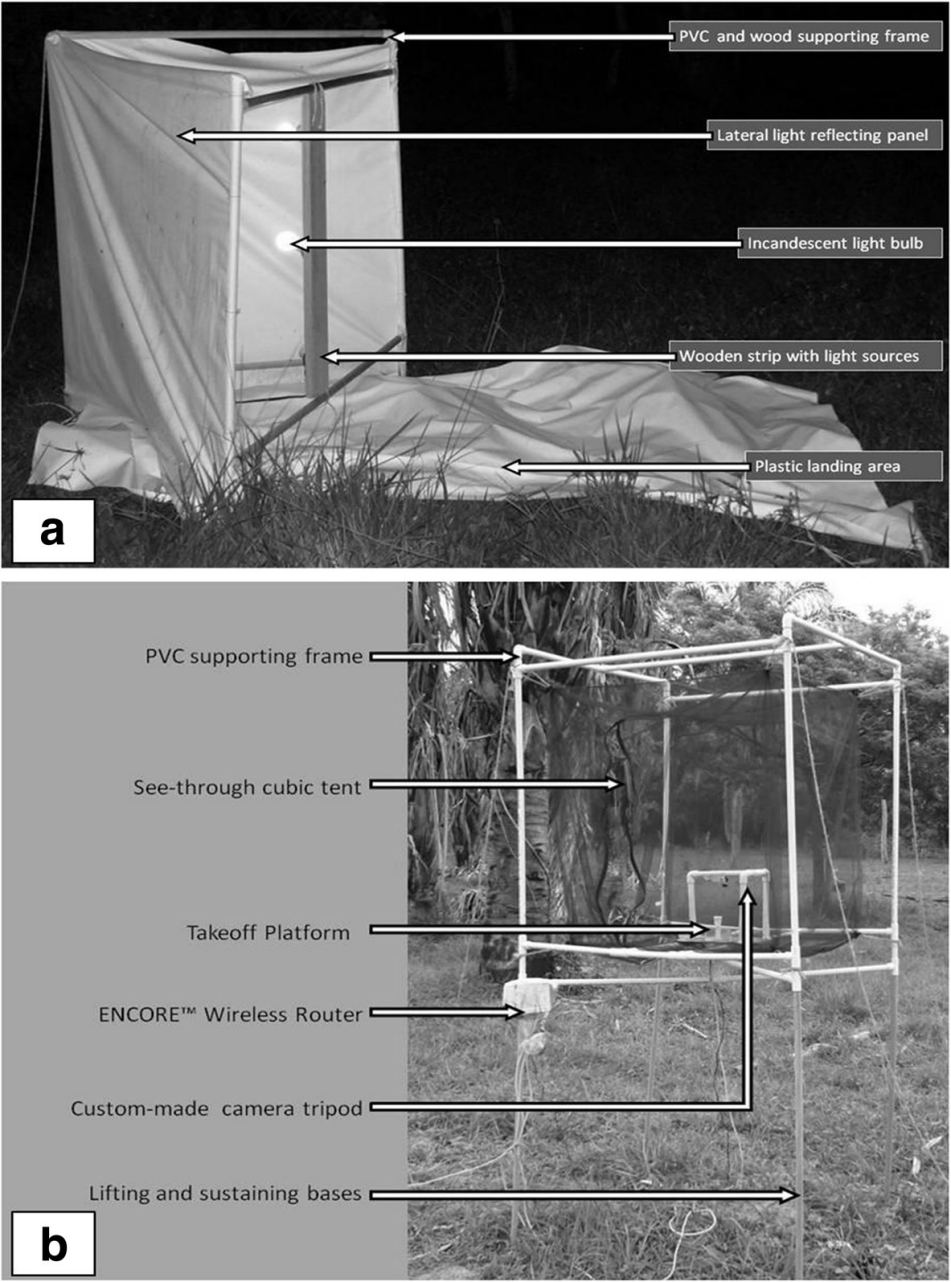
Light traps and take-off arena used in the field experiments. **a**. Directional light traps (modified from Sjorgen & Rickman (1966) [27]). **b**. Experimental cubical tent for take-off experiments (modified from Minoli & Lazzari (2006) [31])

Directional light traps modified from Sjogren and Ryckman (1966) were employed [27]. Each trap consisted of two white plastic “Black Out” panels (1.20 m × 1.00 m). A frame with PVC pipes (1.27 cm in diameter) and wood sustained both panels at right angles on a white plastic landing area (1.40 m × 2.00 m). Three 100 Ws incandescent light bulbs attached to a wooden strip (0.8 m × 0.1 m) and facing inward were used as light sources (Fig. 2a). Two traps that were counterpoised and alternated between four directions (*i.e.*, first night in NE and SW directions, next night NW and SE directions) to determine the dispersal rates and the approximate origins of the flying triatomines during thirty-four consecutive nights. At the end, each one of the four directions was sampled with a directional trap for 17 nights. The panels and external walls of the house were revised every 15 min between 18:45 h and 00:30 h, resulting in a total of 102 h/light trap or 408 h/four light traps sampled.

Live bait and directional light traps were used simultaneously but always preventing interference between both sampling methods.

Insects attracted to the directional light traps were captured manually, identified following keys [4], and those identified as *R. prolixus* were used in later take-off experiments.

### Take-off experiments

Two experimental arenas modified from Minoli and Lazzari (2006) were used [31]. Each arena was a 1 m^3^ tent made of black cloth and elevated 1 m above the ground by a PVC pipe (1.27 cm in diameter) frame (Fig. 2b). Cubic tents were always located to the north of the house at 60 m and 110 m (see “T” on Fig. 1). Inside each cubic tent was a take-off platform (Plexiglass dish 15 cm in diameter and 5 cm high with walls covered in Vaseline) that tested the nightly take-off directions of ten *R. prolixus* (five males and five females) that were marked individually with white body paint. The triatomines were only able to leave the take-off platform by flying from a circular piece of cardboard (3 cm in diameter) marked with eight 45° sectors. The behavior of *R. prolixus* in each cubic tent was recorded with a Genius IP Secure 300R infrared camera supported with a tripod (PVC pipes 1.27 cm in diameter) 15 cm above the take-off platform (Fig. 2b). Each camera was connected to an Encore Electronics 802.11 g router and a Dell Inspirion 5100 computer by 5e Ethernet LAN cables. Lastly, real-time recordings of each platform were carried out during sixteen nights between 19:00 h and 00:30 h with two alternating light conditions (artificial light bulbs of the house facing to the cubic tents turned on or off). In total, eight nights for each experimental arena under each light condition were recorded. The time and take-off sector were registered for each insect.

### Environmental parameters

Temperature, relative humidity and wind velocity were recorded with data loggers (HOBO U23-001 and EXTECH AN400 respectively) every 15 min during all fieldwork nights at 12 m from the house and 2 m above the ground. Low values of environmental night luminosity and the light emitted by the artificial light bulbs of the house were measured manually every 15 min with a generic light dependent resistor (LDR) located 1.6 m above the ground, and 0.3 m away from the researcher. The LDR was connected to a multimeter (UNI-T UT10A), and the results were later converted from Ohms (Ω) to LUX units using the correlation formula *y = 4×10*^*6*^*×*^*-1.47*^ (R^2^ = 0.984). The formula was obtained at dusk during five consecutive days with measurements recorded simultaneously with the LDR-multimeter and a heavy duty light meter (EXTECK 407026).

### Triatomine identification, determination of T. cruzi infection and DTU assignment

The captured sylvatic triatomines were identified using the keys of Lent and Wygodzinsky (1979) [4]. *Rhodnius prolixus* identifications were confirmed by analyzing DNA sequences of the cytochrome b gene [45]. Natural infection with *T. cruzi* was only assessed in the adults that were still alive after the fieldwork. The hindguts of twenty *R. prolixus* (eleven males, nine females) captured in palm trees, and twenty-six *R. prolixus* (twelve males, fourteen females), two *P. geniculatus* males and one *T. maculata* female captured with the directional light traps were examined with a light microscope. The positive feces were transferred to biphasic culture media for parasite isolation. When the isolate reached the logarithmic phase, aliquots of 200 μL were subjected to DNA extraction using the mini-prep Qiagen kit (Qiagen, Barcelona, Spain). The DTU assignment was performed using mitochondrial Multilocus Sequence Typing (MLSTmt) and Multilocus Microsatellite Typing (MLMT) using reference strains with previously established conditions [46].

### Data analysis

ESRI ArcGIS v.9.3 was used to locate the sampled palm trees and the number of triatomines captured with the live bait traps from Google Maps images. Kolmogorov-Smirnov tests for normality were carried out. The effect of environmental parameters on the arrival of triatomines to the light traps was analyzed by linear regressions. Differences in the sex ratios and between the numbers of insects captured in each light trap were tested by the Mann–Whitney U test and the Kruskal-Wallis test, respectively. The Friedman test was used to compare the amount of light reaching each cubic tent with the two light conditions [47]. All the afore-mentioned statistical analyses utilized the SPSS v.16 software. The numbers of insects captured in the directional light traps were analyzed by the Kuiper and Hodges-Ajne test [48] that were calculated with the ORIANA v.3 (http://www.kovcomp.co.uk/oriana/) and Matlab v.8.3, R2014a (http://www.mathworks.com/products/matlab/) software. Friedman test was used to compare the number of insects that began flights at different time periods in the take-off experiments [47]. Take-off directions were analyzed by the Kuiper and Hodges-Ajne test [48] using ORIANA v.3 and Matlab v.8.3, R2014a software.

## Results

### Palm tree sampling, infestation map and *T. cruzi* infection

Four sylvatic species (2169 insects: 2039 in palm trees and 130 actively dispersing) were collected during our field work: *Panstrongylus geniculatus* (0.32 %), *Psammolestes arthuri* (0.18 %), *Triatoma maculata* (0.28 %) and *Rhodnius prolixus* (99.22 %). Most of our results will refer to *R. prolixus* because of its increased abundance over the other triatomine species. Higher concentrations of palm trees with triatomine infestations were found to the north of the house (Fig. 1). Of the ninety-four palm trees sampled, only nine (9.6 %) were negative for triatomines (six *A. butyracea*, two *A. aculeata* and one *C. nucifera*). *R. prolixus* was the most abundant species with 2034 insects captured in palm trees (543 nymphs I (NI), 610 NII, 441 NIII, 183 NIV, 179 NV, 34 males and 44 females). The number of insects captured ranged from one insect/night in five palm trees to 246 insects/night in another palm tree. The mean value and range of the different stages of *R. prolixus* captured per palm tree are shown in Table 1. In addition to *R. prolixus*, *T. maculata* individuals were also captured in palm trees (five NII in two *A. aculeata* and two *A. butyracea*). Eleven males and nine females were dissected and examined microscopically in the hindgut, of these, three *R. prolixus* (two males and one female) were found to be positive for *T. cruzi* (15 %).

**Table 1:** Mean and range of the different stages of *Rhodnius prolixus* captured with live bait in the 94 palm trees in Miramar farm (Paz de Ariporo-Casanare)

Stage	Mean	Minimum	Maximum	St.Dev
N I	8.35	1	80	15.4
N II	9.24	1	97	14.7
N III	5.96	1	42	7.6
N IV	3.59	1	14	3.4
N V	4.26	1	34	5.5
Male	1.62	1	3	0.7
Female	1.57	1	4	0.8

### Directional light trap experiments and *T. cruzi* infection

One hundred and thirty triatomines were captured with the directional light traps. One hundred and eighteen were identified as *R. prolixus* (59 males and 59 females), seven as *P. geniculatus* (six males and one female), four as *Ps. arthuri* (one male, two females and one not determined), and one female as *T. maculata*. Considering only *R. prolixus*, in average 6.9 insects/night and 3.47 insects/directional light trap were captured.

**Fig. 3:**
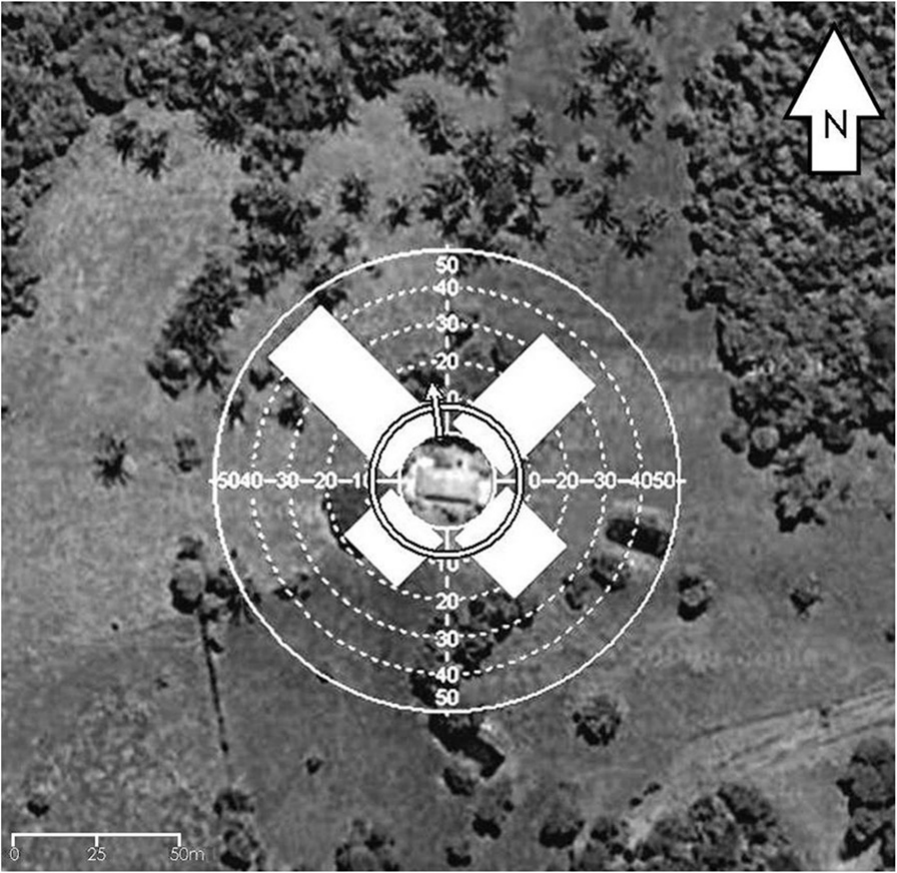
Source of flying *Rhodnius prolixus* captured with directional light traps. Histograms with numbers of *R. prolixus* captured with directional light traps facing to the NW, NE, SW, and SE. White arrow is the mean vector (N = 118 insects, μ = 351.47° and *r* = 0.269). Satellite image from Google Maps®

While the female *T. maculata* was captured on the trap facing NE, the other three species were captured on the traps that faced all four directions sampled. Kuiper’s test showed that our data were not following the von Mises distribution (K > K(α)) [46]. The traps facing to the NE and NW attracted directionally *R. prolixus* (Hodges-Ajne test, *p* < p (α)) (Fig. 3).

**Fig. 4:**
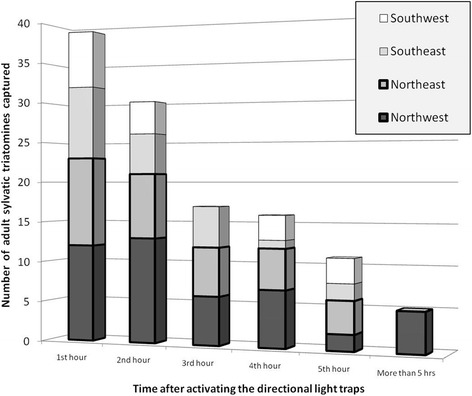
Number of actively dispersing triatomines captured with directional light traps. Histograms with numbers of *R. prolixus* captured per hour after dusk on the directional light traps facing to the NW, NE, SW, and SE

The majority of *R. prolixus* (58.47 %) arrived within the first two hours of sampling (Kruskal-Wallis test, *p* = 0.014) (Fig. 4), and the maximum number of insects captured in one hour was eleven. No significant relationships were found between captures and the environmental parameters measured (Table 2) (temperature *R*^*2*^ *< 0.001, p* = *0.895;* relative humidity *R*^*2*^ *= 0.015, p* = *0.481;* wind speed *R*^*2*^ *= 0.024, p* = *0.452* and night luminosity *R*^*2*^ *= 0.083, p* = *0.122*). The probability of capturing an actively dispersing triatomine on a given night under the range of environmental parameters measured was 0.74. This value was higher during the first two hours after sunset and progressively decreased throughout the night (Fig. 4). No walking nymphs were found to reach the plastic landing area of the directional light traps.

**Table 2:** Mean, maximum and minimum values of environmental variables recorded during 34 nights in Casanare-Colombia

Variable		Mean	Minimum	—	Maximum
Temperature	(°C)	26.17	23.1	—	29.6
Relative Humidity	(%)	81.02	51.4	—	98.4
Wind Speed	(m/s)	0.11	0.0	—	0.6
Luminosity	(μLUX)	74.16	26.15^a^	—	8031.01^b^

^a^Moonless dark night

^b^Cloudless and full moon night

None of the twenty-six dissected *R. prolixus* (twelve males and fourteen females) were found with *T. cruzi,* and only two males of *P. geniculatus* and one female of *T. maculata* were found to be infected with the parasite.

### Molecular characterization of *T. cruzi* infections

We obtained two isolates from *R. prolixus* captured in palm trees; two from *P. geniculatus* and one from *T. maculata* captured with the directional traps. The isolates were cloned, and ten biological clones were obtained from each isolate. The results of the high-resolution molecular characterization of the 20 clones from *P. geniculatus* demonstrated the occurrence of mixed infections of TcI (55 %), TcIII (30 %) and TcIV (15 %)*.* In the case of *R. prolixus* and *T. maculata*, all the clones were typed as TcI with a peculiar tailored allelic profile. In phylogenetic reconstructions (data not shown), these clones clustered in a basal clade that was tightly related to the sylvatic isolates obtained from *Alouatta seniculus* (Linnaeus 1766). Those clones identified as TcI were subsequently analyzed by SL-IR, where the presence of TcId was observed (genotype associated to the sylvatic cycle).

### *Attraction of* R. prolixus to external lights for the house

The search for triatomines on the exterior walls of the house yielded an additional 31 *R. prolixus* (17 males and 14 females), two males of *P. geniculatus* and one male of *Ps. arthuri*. No walking nymphs were found to reach the exterior walls of the house during the sampling.

### *Take-off experiments with* R. prolixus

**Fig. 5:**
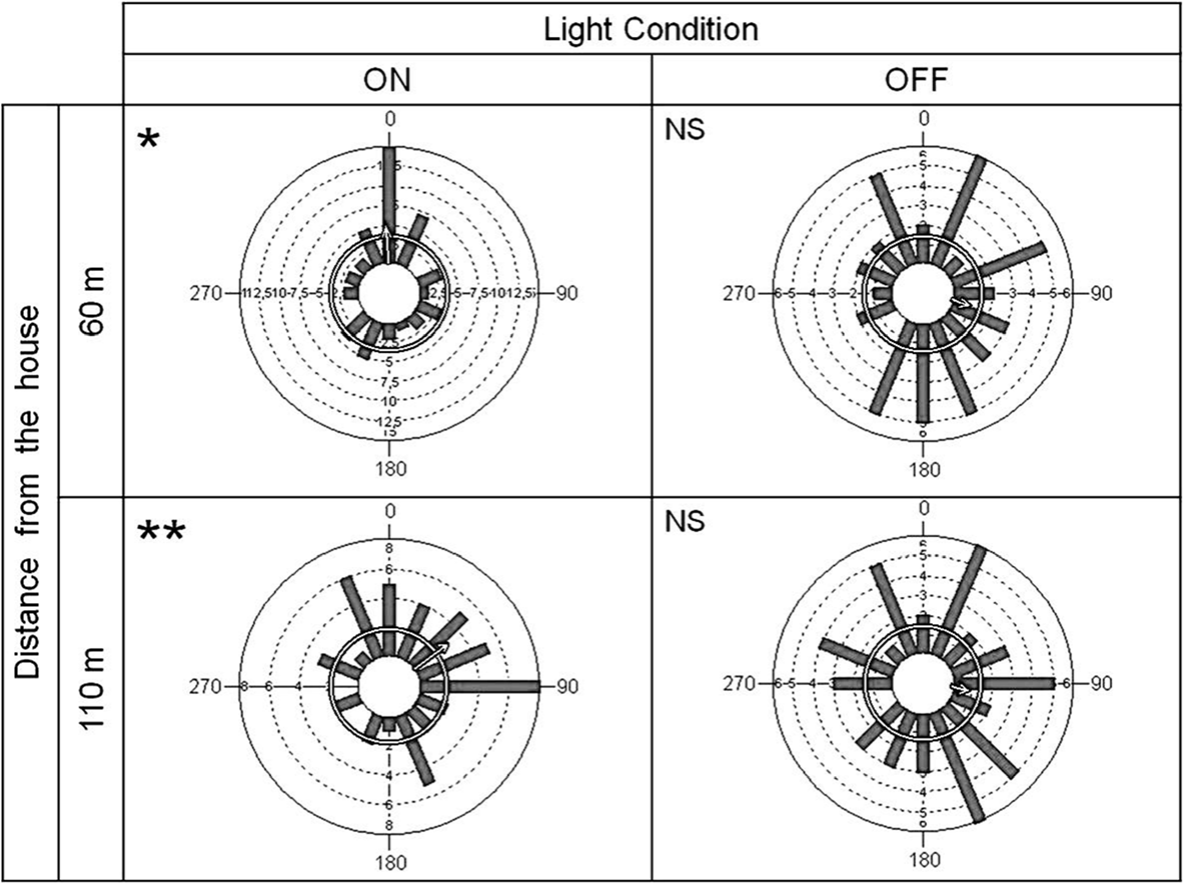
Take-off directions of sylvatic *Rhodnius prolixus*. Histograms with the total numbers of flying *R. prolixus* under field conditions with lights turned on or off. Mean vectors: with light bulbs turned on and at 60 m μ = 356.88°, *r* = 0.306 and at 110 m μ = 52.44°, *r* = 0.358; with light bulbs turned off and at 60 m μ = 101.41°, *r* = 0.135 and at 110 m μ = 96.72°, *r* = 0.125. Asterisks indicates directionality to the house (set at 0°) (Hodges-Ajne test, *p* < p(α)). NS = not significant

On average, 6.5 *R. prolixus* per night initiated flights in each experimental arena. However, the majority (77 %) tended to start flights within the first half hour of the experiments (Friedman test, *p* = 0.001). The take-off by *R. prolixus* was dependent of the natural light conditions and distances of the cubic tents (Friedman test, *p* = 0.02) (Table 3). Both sexes started their flights in similar numbers (Mann–Whitney U test, *p* = 0.087). Take-offs were non directional when the artificial lights of the house were turned off (Hodges-Ajne test, *p* > α cubical tent at 60 m and 110 m). However, directionality to the house (0° in Fig. 5) was found when the lights were turned on (Hodges-Ajne test, *p* < α cubical tent at 60 m and 110 m).

**Table 3:** Average amount of light reaching the take-off experimental arenas under the two light conditions

Light Condition	Distance From the House (m)	Average Light From the House* (μLUX)
ON	60	2237.28
	110	169.68
OFF	60	79.43
	110	40.51

*Friedman test showed statistically significant differences (*p* = 0.02)

## Discussion

The above results, and those reported by Angulo et al. (2012) and Rendon et al. (2015) [41, 49], confirm the relevance of active dispersal by sylvatic triatomines in Colombian and Venezuelan grassland plains, which has been highlighted by a committee of experts [1].

Casanare Department encompasses an area of 44,500 km^2^ with a very low population density (7.44 persons/km^2^) dispersed in rural areas [50]. In rural areas, people usually live in very similar settings: few isolated houses surrounded by palm trees of *Attalea butyracea* [44, 49, 50]. Two advantages were considered for sampling the model house in the present study: Firstly, a high concentration of palm trees to the north side of the house and the presence of a grassland space in between that allowed us to carry out the take-off experiments. Secondly, since the nearest house in the area was located not closer than one km away, we could be certain that no other chemical and visual stimuli from nearly houses interfered with our experiments.

Similar results considering sylvatic dispersion of triatomines could be expected in other municipalities of Casanare considering the above mentioned distribution of the rural population, the similarity in the ecological conditions of the rural areas in the Department [50], and the high prevalence of *Trypanosoma cruz*i infection in Casanare [42].

However, some questions remain to be investigated in Casanare considering potential effects on the attraction of triatomines to artificial light sources. For instance: what will be the effect of several houses together with artificial light sources of different intensities? How would the outcome of the experiments change if tree palms surround houses in closer proximity? And, what should be expected if *A. butyracea* natural forest is replaced by oil tree palm plantations surrounding the houses?

All four sylvatic species of triatomines captured here have been previously reported in Colombian northeastern plains and are associated with sylvatic habitats in Ecuador, Brazil and other South American countries [4, 5, 51, 52]. These species can be divided into three groups based on their risk of domiciliation: *Rhodnius prolixus* and *T. maculata* are frequently domiciliated in Colombia [5], *P. geniculatus* is highly associated with burrows of mammals, tree bark, palm trees and bat caves [4] as well as several domiciliated populations in Colombia and Venezuela [53, 54], and, finally, *Ps. arthuri* which has only been reported in sylvatic niches associated with bird nests, palm trees and the bark of dead trees [4].

Our live bait traps mainly captured *R. prolixus* in palm trees, confirming that *A. butyracea* is a relevant sylvatic niche for *R. prolixus* [5, 41, 49]. Furthermore, the presence *R. prolixus* infected with *T. cruzi* (TcId genotype) in palm trees highlights this active focus of sylvatic *T. cruzi* transmission that is widely distributed in Colombian northeastern plains [5]. Likewise, molecular epidemiology studies in the area have identified *T. cruzi* sylvatic genotypes in *R. prolixus* captured in *Attalea* palms, suggesting that these triatomines represent a high risk factor when invading domestic foci [11, 46, 55]. Moreover, different cohort studies have incriminated *T. cruzi* sylvatic genotypes in cardiomyopathies of chronic patients from Argentina and Colombia [56, 57].

Our results of actively dispersing triatomines infected with *T. cruzi* also show that sylvatic Chagas disease transmission can take place with the aid of *P. geniculatus* in the region. We obtained mixed infections of DTUs in the captured *P. geniculatus* specimens. The DTUs herein reported (TcI, TcIII and TcIV) are closely related to the sylvatic cycle of parasite transmission. The transmission dynamics of these genotypes is maintained by sylvatic reservoirs, such as *Alouatta seniculus, Philander frenata* (Olfers 1818)*, Monodelphis brevicaudata* (Erxleben 1777) and *Dasypus novemcinctus* Linnaeus 1758 [51]. The presence of *T. cruzi* DTUs associated with the sylvatic cycles could increase the probability of new symptomatic cases of Chagas disease in the region. This unorthodox assumption is supported by the findings of TcIII and TcIV in Colombian cardiomyopathic patients where *P. geniculatus* insects have been captured [57]. Additionally, the occurrence of this DTU has been attributed to the emergence of Chagas disease in Venezuela and Bolivia, as vectored by *P. geniculatus* and *T. infestans,* respectively [58, 59].

The high concentration of palm trees with triatomines to the north of the house (Fig. 1) explains the results obtained with the directional light traps. The traps facing to the NE and NW attracted significantly more actively dispersing *R. prolixus* adults than the traps facing to the south (Fig. 3). Field experiments carried out with triatomines suggest that they are able to fly the distances between palm trees, directional light traps and the house [13, 20–23, 28]. The detection of the TcId genotype in *R. prolixus*, which is related to sylvatic habitats, corroborates the notion that the specimens approaching the house initially inhabited the sylvatic niche. The presence of *R. prolixus* infected with sylvatic *T. cruzi* genotypes in palm trees has been previously observed in the Venezuelan grassland plains [60].

To confirm the effect of artificial white light sources [31] on the directionality of active dispersal, we carried out take-off experiments. Our field experiments showing that sylvatic *R. prolixus* of both sexes were induced to fly in response to white light confirmed the importance of artificial white lights for take-off even at 110 m of distance (Table 3 and Fig. 5). The distances tested in our experiments were in the range of the reported flight-dispersal capacity of *Triatoma infestans* [8, 21, 23], suggesting that palm trees located within that radius should be specially regarded following insecticide application in order to reduce colonization or re-colonization. The results herein reported, along with the wide distribution of *Attalea butyracea* in Casanare, could partially explain why 18 % of the houses in Casanare reported re-infestation with *Rhodnius* spp. five months after applying residual insecticides during 2004 and 2005 campaigns [61].

The absence of a correlation between the environmental parameters measured in this study (Table 2) and the high flight dispersals suggests that climatic stability in the grassland plains of Colombia and Venezuela should promote dispersal rates similar to those reported throughout the year, only with a reduction in dispersal on days with heavy rainfall during the day or prior to sunset [27, 32, 33]. In those cases, temperatures below 20 °C or strong air movements in the initial hours after dark will negatively affect triatomine flights [30].

Our findings of actively dispersing *P. geniculatus* and *Ps. arthuri* highlight the importance of other sylvatic species in future control measures against domiciliated triatomines in Colombian northeastern plains. Control measures in Brazil have highlighted the importance of sporadic or progressive invasions of human dwellings by triatomine species that are considered to be secondary vectors after the elimination of the primary vector species [15]. *P. geniculatus* is recognized for its attraction to artificial lights [4, 33], its relationship with *T. cruzi* reservoirs, such as armadillos [4], its infection with *T. cruzi* and its potential risk of domiciliation [53, 54]. The attraction of *Ps. arthuri* to artificial light sources (directional light traps and lighted walls of the house) confirms the findings reported by Angulo et al. (2012) [41] and suggests the attraction to artificial lights of sylvatic species naturally infected with *T. cruzi* even though they are associated to bird nests [4].

Although low numbers of *P. geniculatus* and *T. maculata* were captured in our study, it is important to highlight the infection rates with *T. cruzi* found in both species (2 of 7 individuals of *P. geniculatus* and 1 of 1 of *T. maculata*). Both species should be carefully considered by health authorities for maintaining Chagas *Trypanosoma cruzi* circulation in Casanare, as has been shown in other areas of Colombia [62–64].

The relevance of our findings is highlighted by the acute oral outbreak of Chagas disease that occurred recently in the town of Paz Ariporo (located at approximately 4 Km of our field study area), affecting 31 workers from companies linked to the mining sector who were exposed to food contaminated with either traces or the feces of infected triatomines [49].

How can our results help to reduce the active dispersal of sylvatic triatomines in grassland plains ecosystems? One recommendation is that external artificial lights on walls remain turned off for the first hours after sunset, which is when most sylvatic triatomines find favorable atmospheric and environmental conditions for dispersal (Fig. 4). It has previously been reported that triatomines are captured at higher rates within the first hours after sunset [12, 17, 27, 30]. This recommendation, which sounds very promising, should be tested by an 'adaptive management' strategy to see if this policy would be effective in practice.

## Conclusions

Our results using a model house from the northeastern Colombian plains confirm that four triatomine species (*Rhodnius prolixus*, *Triatoma maculata*, *Psammolestes arthuri* and *Panstrongylus geniculatus*) are actively dispersing in the area and that they are highly attracted to artificial lights. Furthermore, the environmental parameters encountered during this study, particularly during the first hours after sunset, are favorable for the active dispersal of sylvatic triatomines. Finally, sylvatic *R. prolixus* initiates flight independently of sex, night luminosity, and the quantity of artificial light. However, the directionality of the take-offs is determined by the artificial light stimuli and the distance to its source. Our results suggest that the absence of systematic surveillance of dispersing sylvatic triatomines in Colombian grassland plains leads to underestimates of the risk that these species represent for chemical control campaigns and for the introduction of sylvatic *T. cruzi* DTUs into the domestic environment. The increasing likelihood that sylvatic DTUs influence the development of cardiomyopathy highlights the need to pursue further studies in the region aimed at establishing robust surveillance programs. Such programs will hopefully prevent the emergence of secondary Chagas disease vectors in Colombia.

## Abbreviations

cm: centimeter
DTUs: discrete typing units
h: hour
km: Kilometers
LDR: light dependent resistor
m: meter
m.a.s.l.: meters above sea level
MLMT: Multilocus Microsatellite Typing
MLSTmt: mitochondrial Multilocus Sequence Typing
N: North
NE: Northeast
NI: first instar nymphs
NII: second instar nymphs
NIII: third instar nymphs
NIV: fourth instar nymphs
NV: fifth instar nymphs
NW: Northwest
SE: Southeast
SL-IR: Spliced Leader Intergenic Region
SW: Southwest
TcI: *Trypanosoma cruzi* Discrete Typing Unit 1 I
TcII: *Trypanosoma cruzi* Discrete Typing Unit 2 II
TcIII: *Trypanosoma cruzi* Discrete Typing Unit 3 III
TcIV: *Trypanosoma cruzi* Discrete Typing Unit 4 IV
TcV: *Trypanosoma cruzi* Discrete Typing Unit 5 V
TcVI: *Trypanosoma cruzi* Discrete Typing Unit 6 VI
Ws: Watts
W: West
μL: microliter
